# Supplementary material for: Nitrosative stress under microaerobic conditions triggers inositol metabolism in Pseudomonas extremaustralis
Source: PLoS One. 2024 May 2;19(5):e0301252. doi: 10.1371/journal.pone.0301252 (PMC11065229; doi:10.1371/journal.pone.0301252)
Supplement: S1 Fig — Quality summary of sequencing data (a) and dot plot representing normalized counts for each RNAseq replicate in microaerobic culture conditions (b) and microaerobic culture subjected to GSNO (m-NS) (c). (PDF) [file pone.0301252.s001.pdf]

**a**

| | Raw reads | Clean Reads | Q20 (%) | Q30( %) | GC (%) |
|---|---|---|---|---|---|
| **Microaerobiosis R1** | 14594581 | 14385715 | 94.68 | 92.11 | 53 |
| **Microaerobiosis R2** | 14290257 | 14076623 | 94.06 | 91.46 | 53 |
| **m-NS R1** | 12200535 | 12043019 | 94.77 | 92.21 | 54 |
| **m-NS R2** | 11406689 | 11128669 | 93.75 | 91.03 | 53 |

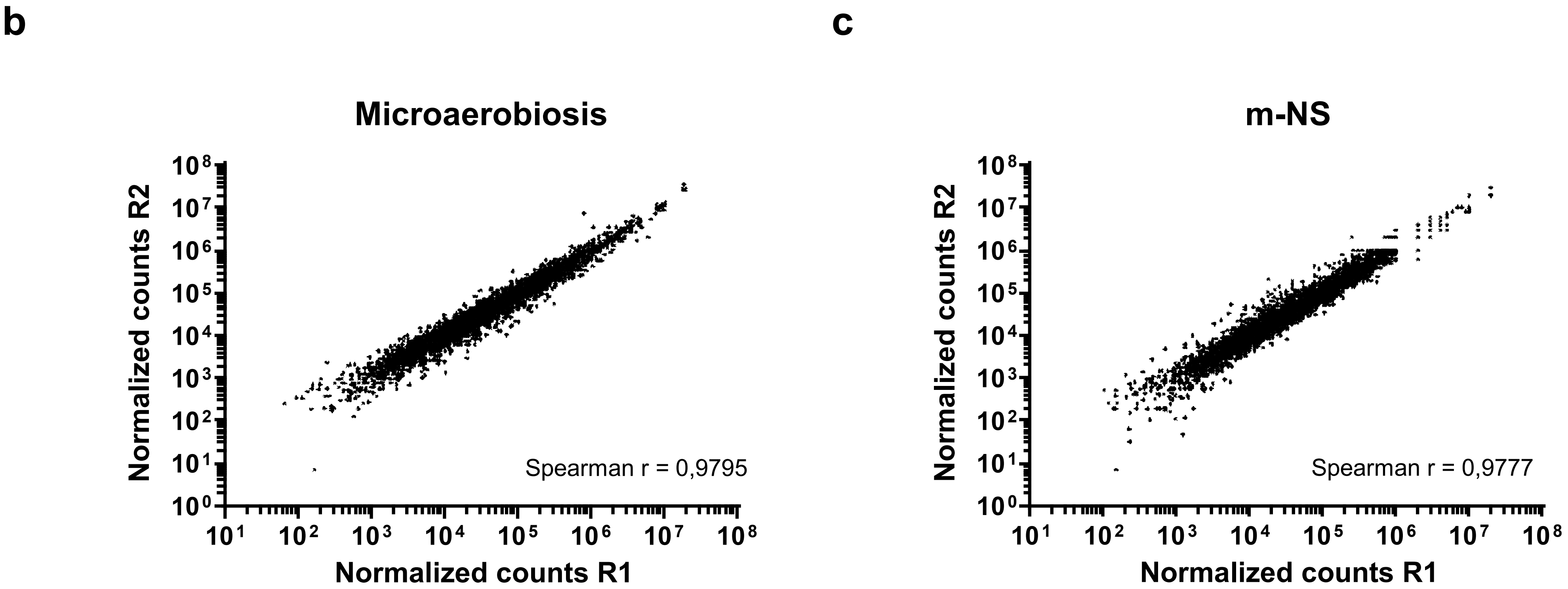


**S1 Fig**. **Transcriptome data information**. Quality summary of sequencing data (a) and dot plot representing normalized counts for each RNAseq replicate in microaerobic culture conditions (b) and microaerobic culture subjected to GSNO (m-NS) (c).
