# Supplementary material for: Nitrosative stress under microaerobic conditions triggers inositol metabolism in Pseudomonas extremaustralis
Source: PLoS One. 2024 May 2;19(5):e0301252. doi: 10.1371/journal.pone.0301252 (PMC11065229; doi:10.1371/journal.pone.0301252)
Supplement: S2 Fig — a. Volcano plots depicting–log10 (Q-value) versus log2 (Fold Change -FC-). Differentially expressed genes (DEGs) are represented by colored dots. Green dots represent upregulated genes (P-value and Q-value <0.05, Fold change >1.5) and red dots represent downregulated genes (P-value and Q-value <0.05, Fold change < -1.5) in presence of GSNO. For the construction of the Volcano plots, genes were initially filtered based on their P-values. All resulting genes were plotted and filtered again by Q-value. b. Quantitative Real Time PCR. Comparative expression analysis of 3 selected genes between m-NS and microaerobic growth conditions. Values represent the mean ± SD of three independent experiments. (PDF) [file pone.0301252.s002.pdf]

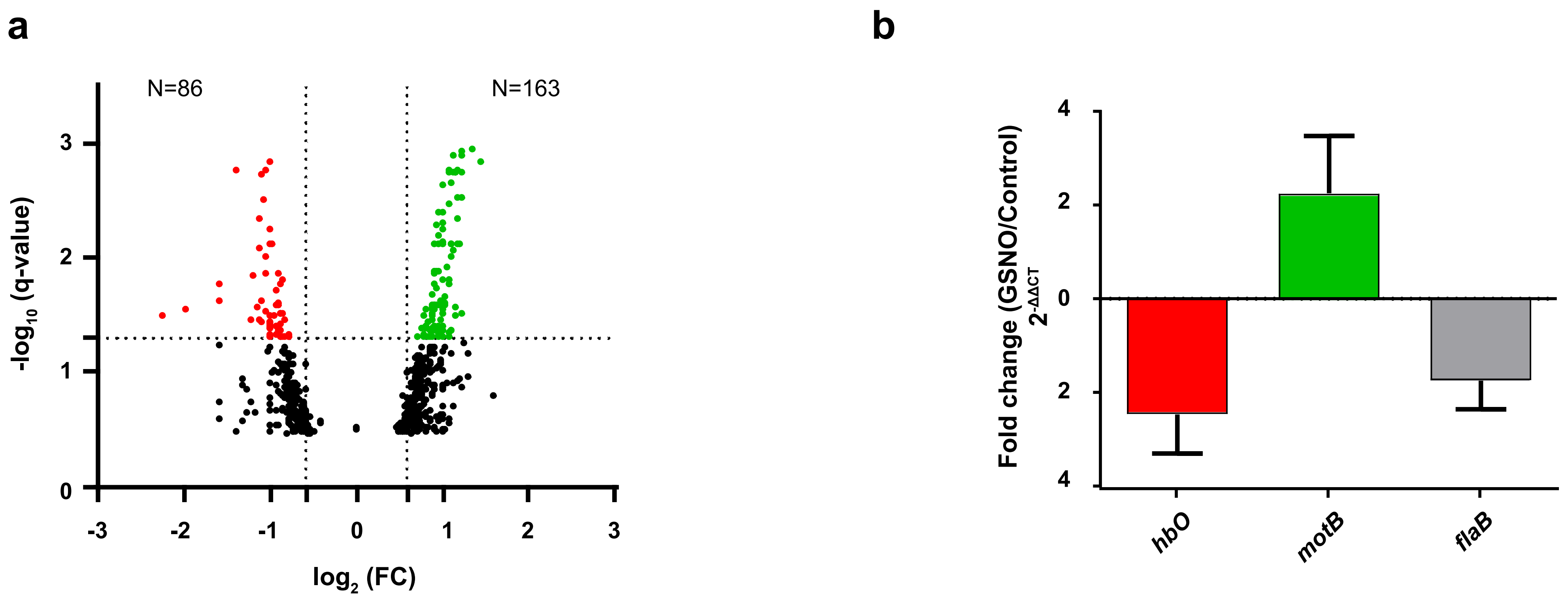

S2 Fig. GSNO effect on gene expression. a. Volcano plots depicting –log10 (Q-value) versus log2 (Fold Change -FC-). Differentially expressed genes (DEGs) are represented by colored dots. Green dots represent upregulated genes (P-value and Q-value <0.05, Fold change >1.5) and red dots represent downregulated genes (P-value and Q-value <0.05, Fold change < -1.5) in presence of GSNO. For the construction of the Volcano plots, genes were initially filtered based on their P-values. All resulting genes were plotted and filtered again by Q-value. b. Quantitative Real Time PCR. Comparative expression analysis of 3 selected genes between m-NS and microaerobic growth conditions. Values represent the mean ± SD of three independent experiments..
