# Supplementary material for: Nitrosative stress under microaerobic conditions triggers inositol metabolism in Pseudomonas extremaustralis
Source: PLoS One. 2024 May 2;19(5):e0301252. doi: 10.1371/journal.pone.0301252 (PMC11065229; doi:10.1371/journal.pone.0301252)
Supplement: S3 Fig — Flow cytometry assays using Mercury Orange in cultures with glucose (G) or myo-inositol (MI) as the sole carbon source, with or without GSNO. a. Geometric mean (Gm) for Mercury Orange fluorescence b. Proportion of cells which present positive signal for Mercury Orange, calculated using a non-stained control. (PDF) [file pone.0301252.s003.pdf]

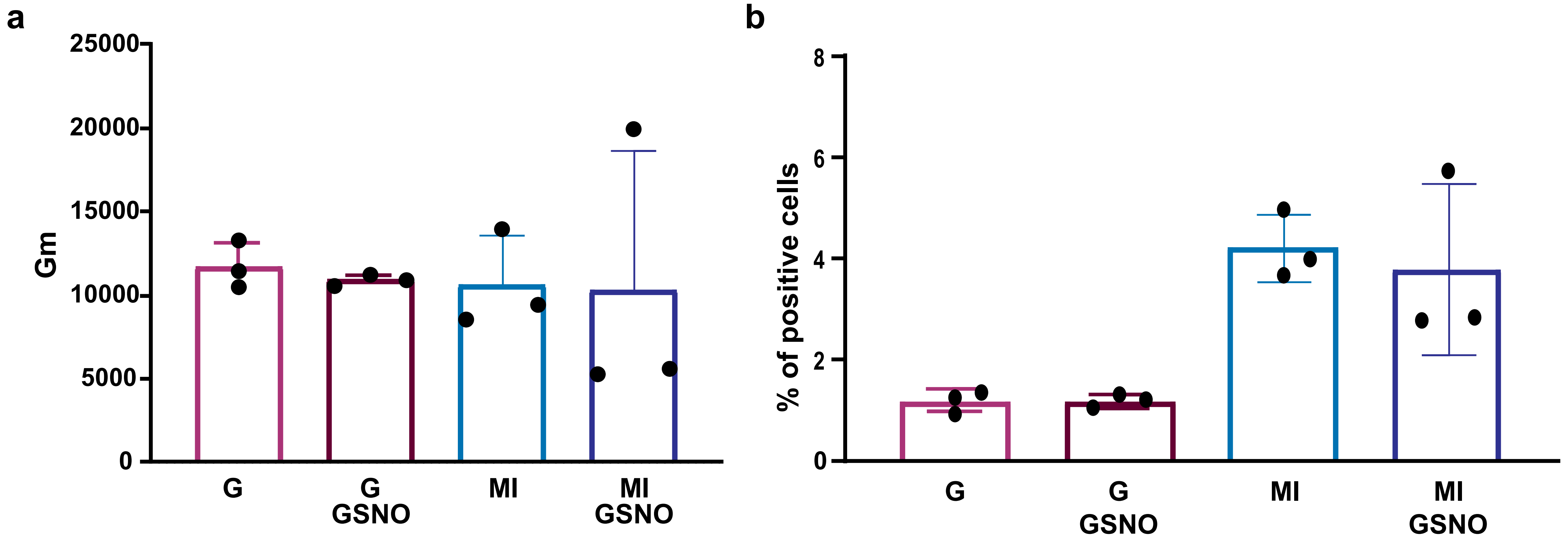

S3 Fig. GSNO effect on the reduced thiol pool. Flow cytometry assays using Mercury Orange in cultures with glucose (G) or *myo*-inositol (MI) as the sole carbon source, with or without GSNO. a. Geometric mean (Gm) for Mercury Orange fluorescence b. Proportion of cells which present positive signal for Mercury Orange calculated using a non-stained control.
