# Supplementary material for: Nitrosative stress under microaerobic conditions triggers inositol metabolism in Pseudomonas extremaustralis
Source: PLoS One. 2024 May 2;19(5):e0301252. doi: 10.1371/journal.pone.0301252 (PMC11065229; doi:10.1371/journal.pone.0301252)
Supplement: S4 Fig — Scheme of the experimental design used to study the resistance to nitro-oxidative stress derivate from the combination of the nitrite accumulated and H2O2. (PDF) [file pone.0301252.s004.pdf]

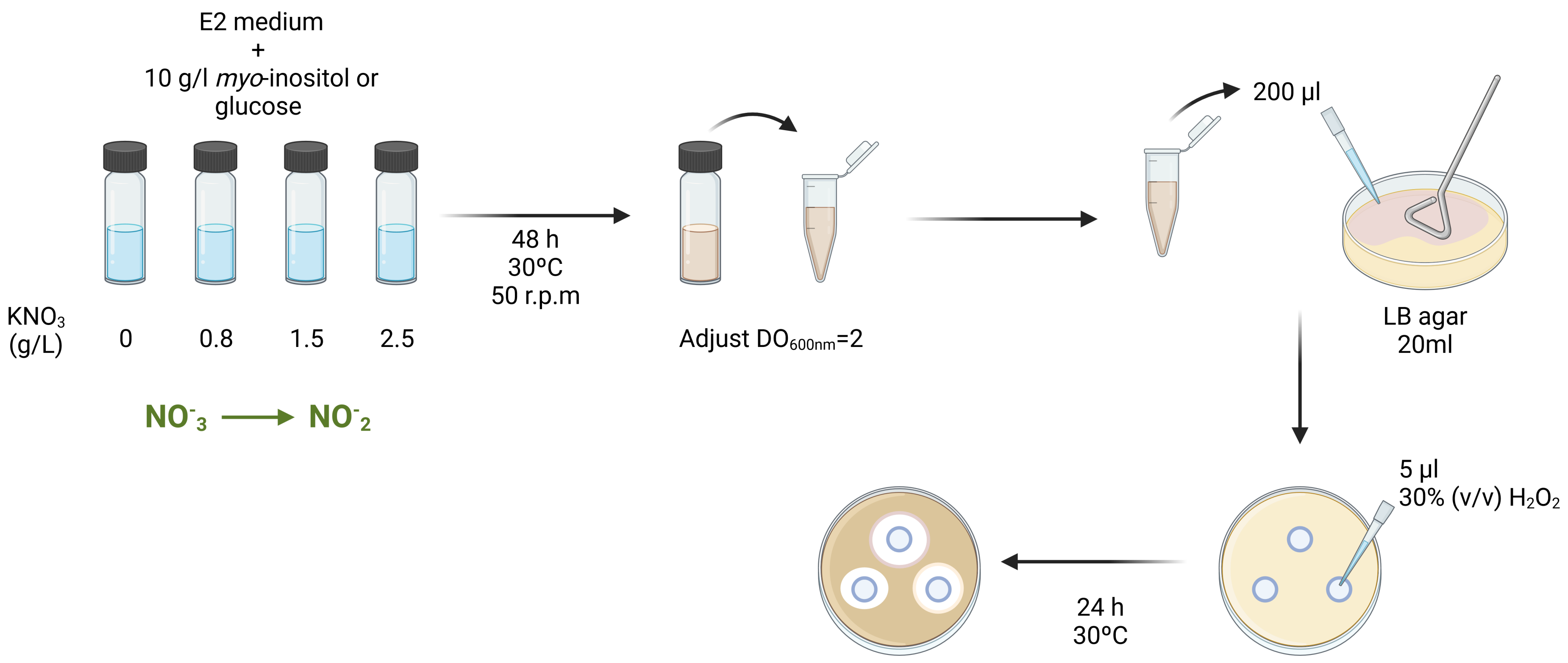

S4 Fig. Nitro-oxidative stress assay. Scheme of the experimental design used to study the resistance to nitro-oxidative stress derivate from the combination of the nitrite accumulated and H_2O_2.
